# Supplementary material for: Predicting Cardiovascular Disease Mortality: Leveraging Machine Learning for Comprehensive Assessment of Health and Nutrition Variables
Source: Nutrients. 2023 Sep 11;15(18):3937. doi: 10.3390/nu15183937 (PMC10534618; doi:10.3390/nu15183937)
Supplement: Supplementary file 1 [file nutrients-15-03937-s001.zip › nutrients-2584112-supplementary.pdf]

Table S1. List of all variables included in the health model

Variable name	Definition
Age	Age (years)
Alcohol	Alcohol habits (1 if drink, 2 if quit, 3 if never)
BMI	Body mass index (kg/m^2)
Ca	Blood calcium (mg/dL)
Dia BP	Diastolic blood pressure (mmHg)
Diabetic	Doctor told you have diabetes (1 if yes or borderline, 0 if no)
Education	Education level (1 if less than high school, 2 if high school, 3 if some college/AA degree, 4 if college graduate or above)
eGFR	Estimated glomerular filtration rate (mL/min/1.73 m^2)
Fe	Blood iron (ug/dL)
GlycoHb	Glycohemoglobin (%)
Hb	Hemoglobin (g/dL)
HDL	HDL cholesterol (mg/dL)
Health ins	Covered by health insurance (1 if yes, 2 if no or unknown)
K	Blood potassium (mmol/L)
LDH	Lactate dehydrogenase (U/L)
Men Health	Days of bad mental health in the last 30 days (days)
Na	Blood sodium (mmol/L)
P	Blood phosphorus (mg/dL)
Phy Health	Days of bad physical health in the last 30 days (days)
Race	Race (1 if Hispanic or other, 2 if non-Hispanic Black, 3 if non-Hispanic White)
Sex	Sex (1 if male, 2 if female)
Smoker	Smoking habits (1 if smoker, 2 if quit, 3 if never)
Sys BP	Systolic blood pressure (mmHg)
Total Cho	Blood total cholesterol (mg/dL)
Uric acid	Blood uric acid (mg/dL)
Waist	Waist (cm)

Table S2. List of all variables included in the nutrition model

Variable name	Definition
Alc int	Alcohol intake (g)
Ca int	Calcium (mg)
Caff int	Caffeine intake (mg)
Cho int	Cholesterol intake (mg)
Cu int	Copper intake (mg)
Fe int	Iron intake (mg)
Fib int	Dietary fiber (g)
Fish int	Eaten fish in the past 30 days (1 if yes, 2 if no/refused/unknown)
Fol int	Total folate intake (µg)
K int	Potassium intake (mg)
MFA int	Total monounsaturated fatty acids intake (g)
Mg int	Magnesium intake (mg)
Milk int	Milk intake (1 if whole, 2 if low fat, 3 if skim, 4 if no or very low milk)
Na int	Sodium intake (mg)
Nia int	Niacin intake (mg)
PFA int	Total polyunsaturated fatty acids intake (g)
Prot int	Protein intake (g)
Salt freq	How often addsalt to food at table (1 if rarely, 2 if occasionally, 3 if very often)
Salt prep	Salt usage in food preparation (1 if never or rarely, 2 if occasionally, 3 if very often)
Salt table	Adds salt at the table (1 if yes, 0 if no)
SFA int	Total saturated fatty acids intake (g)
Sug int	Total sugars intake (g)
Theo int	Theobromine intake (mg)
Vit A int	Vitamin A intake (µg)
Vit B1 int	Thiamin (Vitamin B1) intake (mg)
Vit B12 int	Vitamin B12 intake (µg)
Vit B2 int	Riboflavin (Vitamin B2) intake (mg)
Vit B6 int	Vitamin B6 intake (mg)
Vit C int	Vitamin C (mg)
Vit E int	Vitamin E as alpha-tocopherol intake (µg)
Vit K int	Vitamin K intake (µg)
Wat int	Total plain water drank (g)
Zn int	Zinc intake (mg)

Table S3. General characteristics of the health model

Variable	Control (n = 9490)	CVD-death (n=216)	P-value
Age, years	43.0 [31.0,57.0]	68.0 [60.0,75.0]	<0.001
Sex			<0.001
Male	4600 (48.5)	148 (68.5)	
Female	4890 (51.5)	68 (31.5)	
Race			0.009
Hispanic and Other race	3082 (32.5)	49 (22.7)	
Non-Hispanic Black	1565 (16.5)	42 (19.4)	
Non-Hispanic White	4843 (51.0)	125 (57.9)	
Health Ins			<0.001
Yes	7525 (79.3)	199 (92.1)	
No	1965 (20.7)	17 (7.9)	
Education			<0.001
Less than 9th grade	2004 (21.1)	72 (33.3)	
High school grade	2049 (21.6)	65 (30.1)	
Some college, AA degree	2817 (29.7)	40 (18.5)	
College grad	2620 (27.6)	39 (18.1)	
Diabetic			<0.001
No	8646 (91.1)	161 (74.5)	
Yes	844 (8.9)	55 (25.5)	
Smoker			<0.001
Smoking	1782 (18.8)	60 (27.8)	
Quit	2207 (23.3)	77 (35.6)	
Never	5501 (58.0)	79 (36.6)	
Alcohol			0.208
Current drinker	1118 (11.8)	32 (14.8)	
Current non-drinker	8372 (88.2)	184 (85.2)	
Phy Health, days	0.0 [0.0,2.6]	0.0 [0.0,4.0]	0.31
Men Health, days	0.0 [0.0,3.0]	0.0 [0.0,3.0]	0.197
BMI, kg/m^2	27.6 [24.2,32.0]	28.3 [25.6,32.1]	0.026
Waist, cm	97.8 (15.3)	104.8 (14.9)	<0.001
Sys BP, mmHg	119.0 [110.0,128.3]	131.0 [122.0,144.0]	<0.001
Dia BP, mmHg	70.7 (10.5)	69.5 (11.5)	0.125
Total Cho, mg/dL	197.7 (36.7)	197.8 (35.7)	0.993
HDL, mg/dL	53.0 (14.1)	51.1 (12.7)	0.032
Hb, g/dL	14.3 [13.4,15.3]	14.6 [13.7,15.2]	0.01
GlycoHb, %	5.4 [5.2,5.7]	5.7 [5.5,6.1]	<0.001
Ca, mg/dL	9.4 (0.3)	9.5 (0.3)	0.162
Fe, ug/dL	86.7 (30.8)	84.0 (28.0)	0.157
P, mg/dL	3.8 (0.5)	3.7 (0.5)	0.005
K, mmol/L	3.9 [3.8,4.1]	4.0 [3.9,4.2]	<0.001
LDH, U/L	127.3 (21.3)	138.5 (20.5)	<0.001
Na, mmol/L	139.0 [138.0,140.0]	139.7 [139.0,140.5]	<0.001
Uric acid, mg/dL	5.3 [4.4,6.1]	5.8 [5.3,6.7]	<0.001
eGFR, mL/min/1.73 m^2	103.3 (26.4)	81.4 (21.9)	<0.001

Numerical values are presented as the mean (standard deviation) if the distribution is approximately normal, or as the median (interquartile range) if the distribution is skewed. For categorical variables, percentages are given. Discrepancies in characteristics were assessed through the implementation of the unpaired Student's t-test, Kruskal-Wallis test, or Chi-squared test.

Table S4. General characteristics of the nutrition model

Variable	Control (n = 9490)	CVD-death (n=216)	P-value
Fish int			0.001
Yes	7198 (75.8)	185 (85.6)	
No	2292 (24.2)	31 (14.4)	
Milk int			0.006
Whole	1838 (19.4)	40 (18.5)	
Low fat	5136 (54.1)	123 (56.9)	
Skim	1141 (12.0)	11 (5.1)	
No/Very low milk	1375 (14.5)	42 (19.4)	
Alc int, g	0.0 [0.0,7.0]	0.0 [0.0,3.8]	0.279
Ca int, mg	882.8 [630.5,1214.4]	726.8 [516.9,959.0]	<0.001
Caff int, mg	101.0 [32.5,212.4]	128.5 [40.1,254.2]	0.027
Cho int, mg	252.5 [165.5,382.5]	270.0 [165.2,380.9]	0.524
Cu int, mg	1.2 [1.0,1.6]	1.1 [0.9,1.4]	<0.001
Fe int, mg	14.7 [11.1,19.5]	13.4 [10.1,17.9]	0.002
Fib int, g	16.2 [11.7,22.4]	13.9 [10.7,18.0]	<0.001
Fol int, µg	389.0 [289.5,528.0]	344.2 [270.2,447.2]	<0.001
K int, mg	2656.2 [2090.1,3367.5]	2509.0 [1948.5,3096.4]	0.004
Mg int, mg	289.0 [225.0,370.5]	260.0 [200.0,312.6]	<0.001
Na int, mg	3335.5 [2548.5,4307.4]	2981.0 [2393.9,3766.9]	<0.001
Nia int, mg	24.1 [18.3,31.5]	21.2 [16.5,27.0]	<0.001
MFA int, g	27.2 [19.5,36.9]	25.4 [18.7,31.1]	0.003
PFA int, g	16.3 [11.6,22.4]	14.0 [9.7,18.4]	<0.001
SFA int, g	24.1 [16.9,33.1]	22.6 [16.1,29.7]	0.014
Prot int, g	80.9 [62.8,102.9]	73.4 [58.8,86.6]	<0.001
Sug int, g	107.8 [74.6,150.5]	87.9 [61.3,130.9]	<0.001
Theo int, mg	12.0 [0.0,46.0]	5.0 [0.0,37.8]	0.008
Vit B1 int, mg	1.6 [1.2,2.1]	1.4 [1.1,1.9]	<0.001
Vit B2 int, µg	2.0 [1.5,2.7]	2.0 [1.5,2.5]	0.143
Vit B6 int, mg	1.9 [1.4,2.6]	1.7 [1.2,2.3]	<0.001
Vit B12 int, µg	5.0 [3.0,7.9]	4.6 [2.9,7.3]	0.338
Vit C int, mg	73.8 [37.5,127.5]	68.4 [32.0,110.8]	0.047
Vit E int, µg	6.9 [4.9,9.9]	5.6 [4.2,7.8]	<0.001
Vit K int, µg	71.0 [44.4,123.3]	57.5 [40.3,97.5]	0.001
Zn int, mg	11.1 [8.2,14.8]	9.9 [7.8,14.1]	0.033
Wat int, g	843.9 [355.5,1480.7]	710.6 [266.6,1306.4]	0.029
Salt freq			0.97
Rarely	5809 (61.2)	131 (60.6)	
Occasionally	2115 (22.3)	48 (22.2)	
Very often	1566 (16.5)	37 (17.1)	
Salt prep			0.041
Never or rarely	2244 (23.6)	67 (31.0)	
Occasionally	3123 (32.9)	66 (30.6)	
Very Often	4123 (43.4)	83 (38.4)	
Salt table			0.562
does not add	2706 (28.5)	66 (30.6)	
Adds	6784 (71.5)	150 (69.4)	

Numerical values are presented as the mean (standard deviation) if the distribution is approximately normal, or as the median (interquartile range) if the distribution is skewed. For categorical variables, percentages are given. Discrepancies in characteristics were assessed through the implementation of the unpaired Student's t-test, Kruskal-Wallis test, or Chi-squared test.

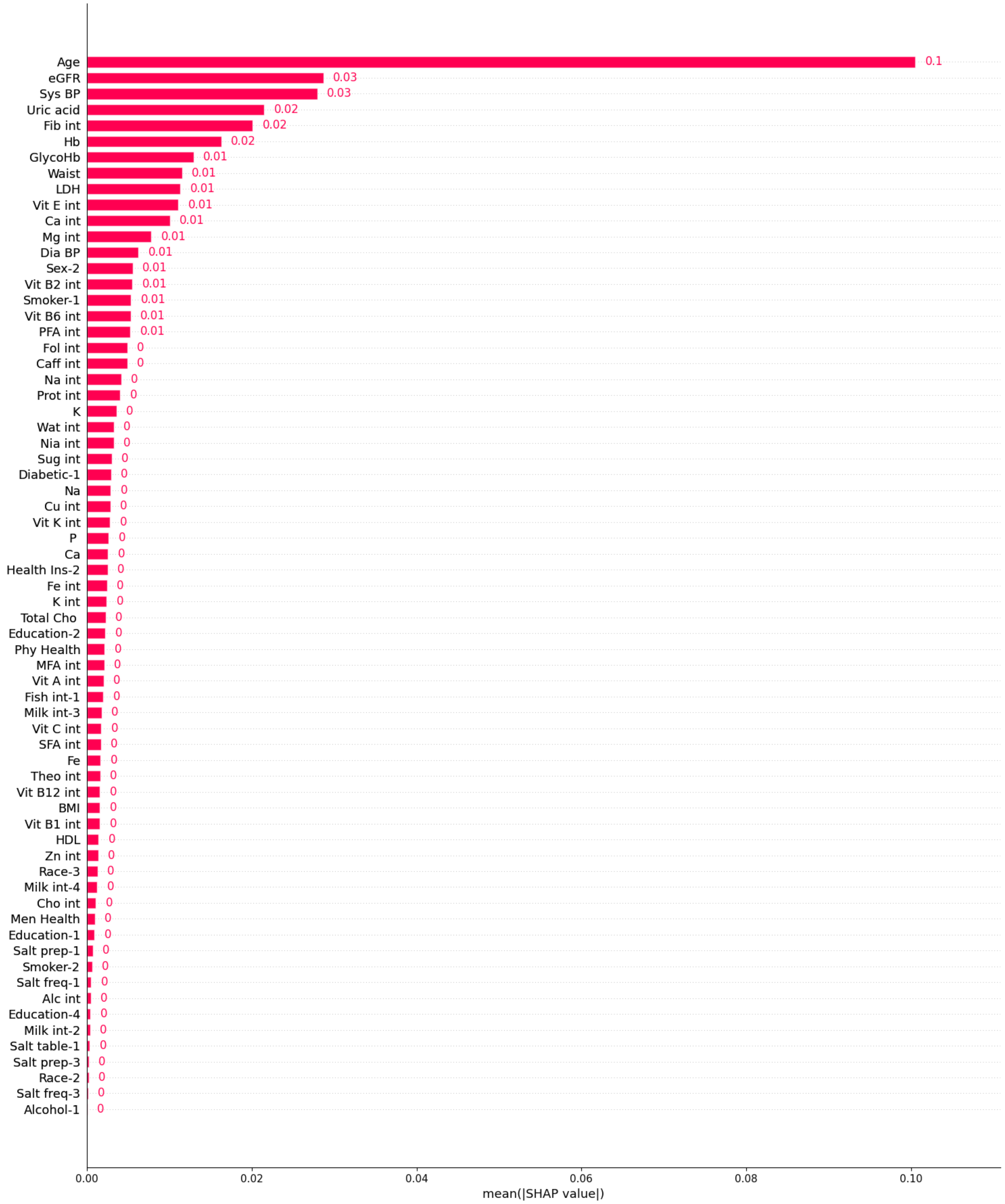

Figure S1. Feature importance from mixed model for cardiovascular disease death based on SHAP value distribution.

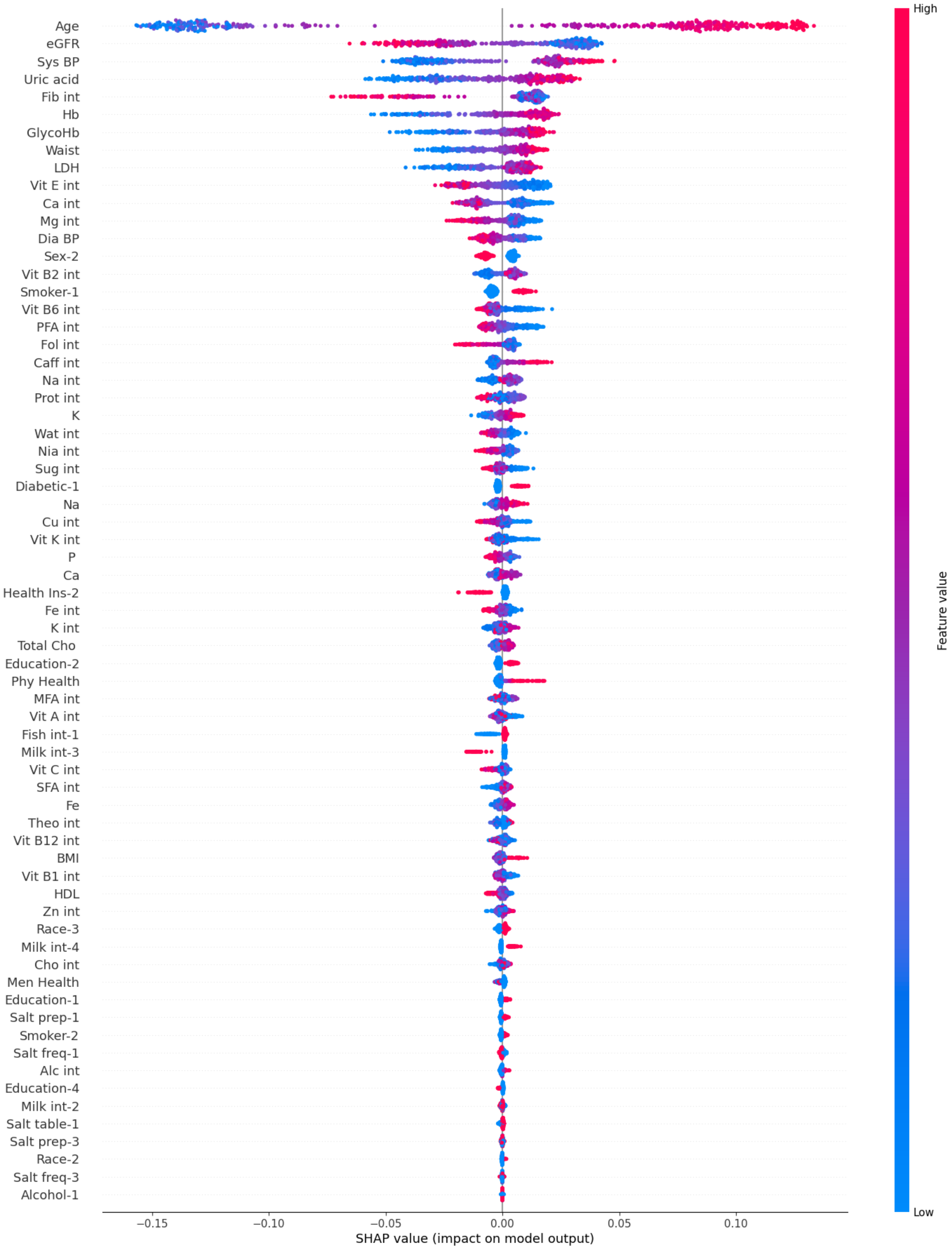

Figure S2. Feature importance from mixed model for cardiovascular disease death based on SHAP distribution values. Positive SHAP-values are indicative of positive correlation with CVD death, while negative SHAP-values are indicative of negative correlation.

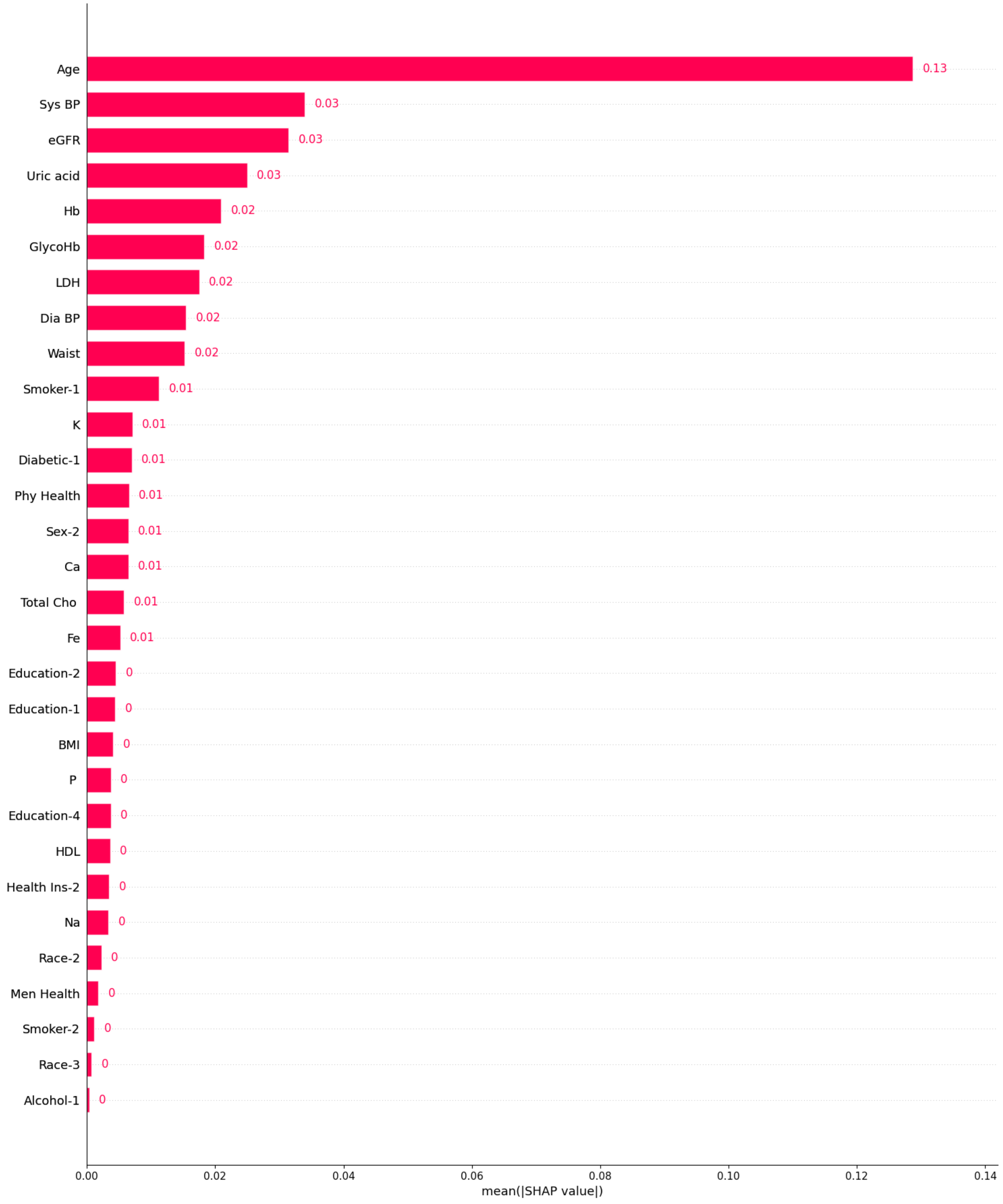

Figure S3. Feature importance from health model for cardiovascular disease death based on SHAP absolute values.

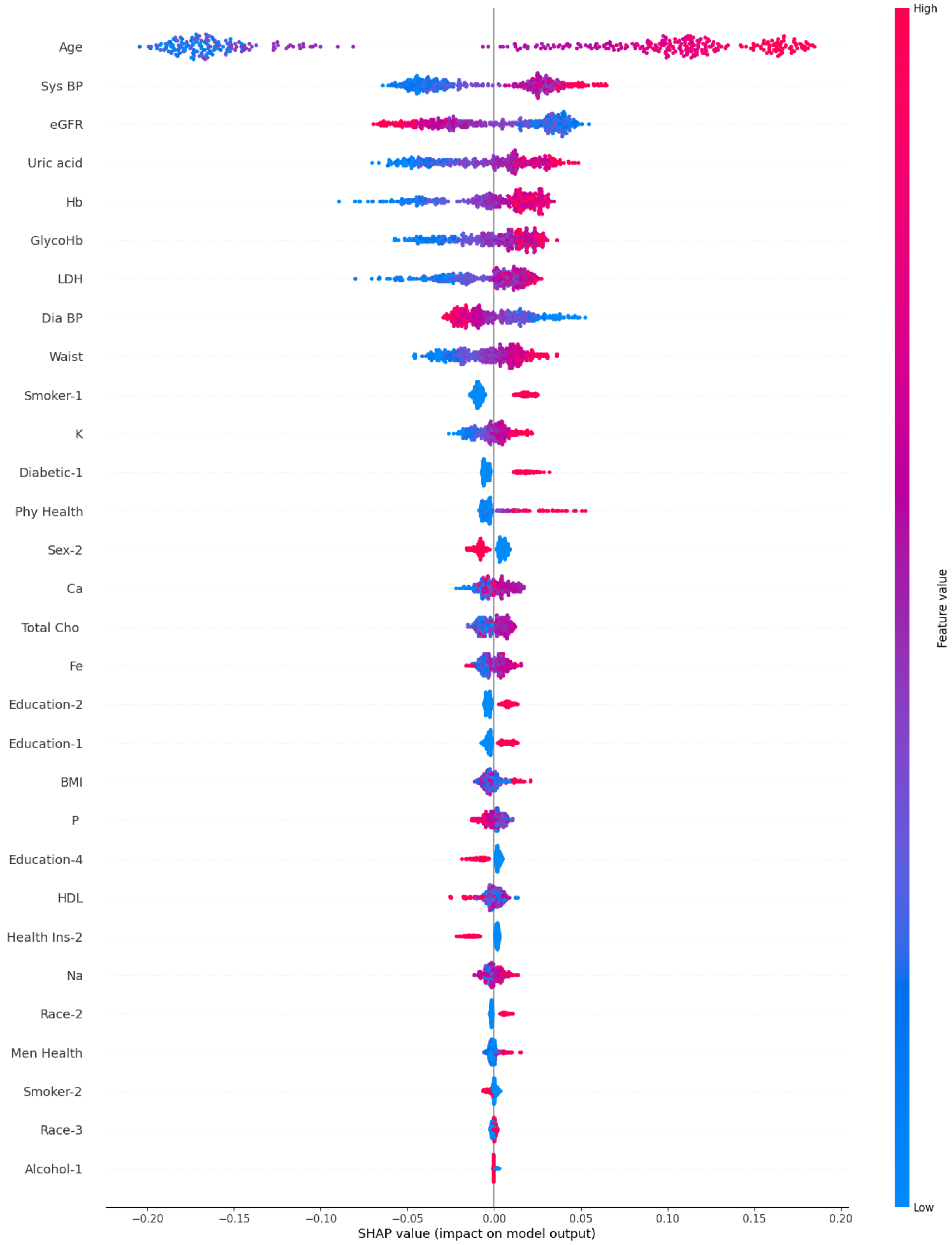

Figure S4. Feature importance from health model for cardiovascular disease death based on SHAP value distribution. Positive SHAP-values are indicative of positive correlation with CVD death, while negative SHAP-values are indicative of negative correlation.

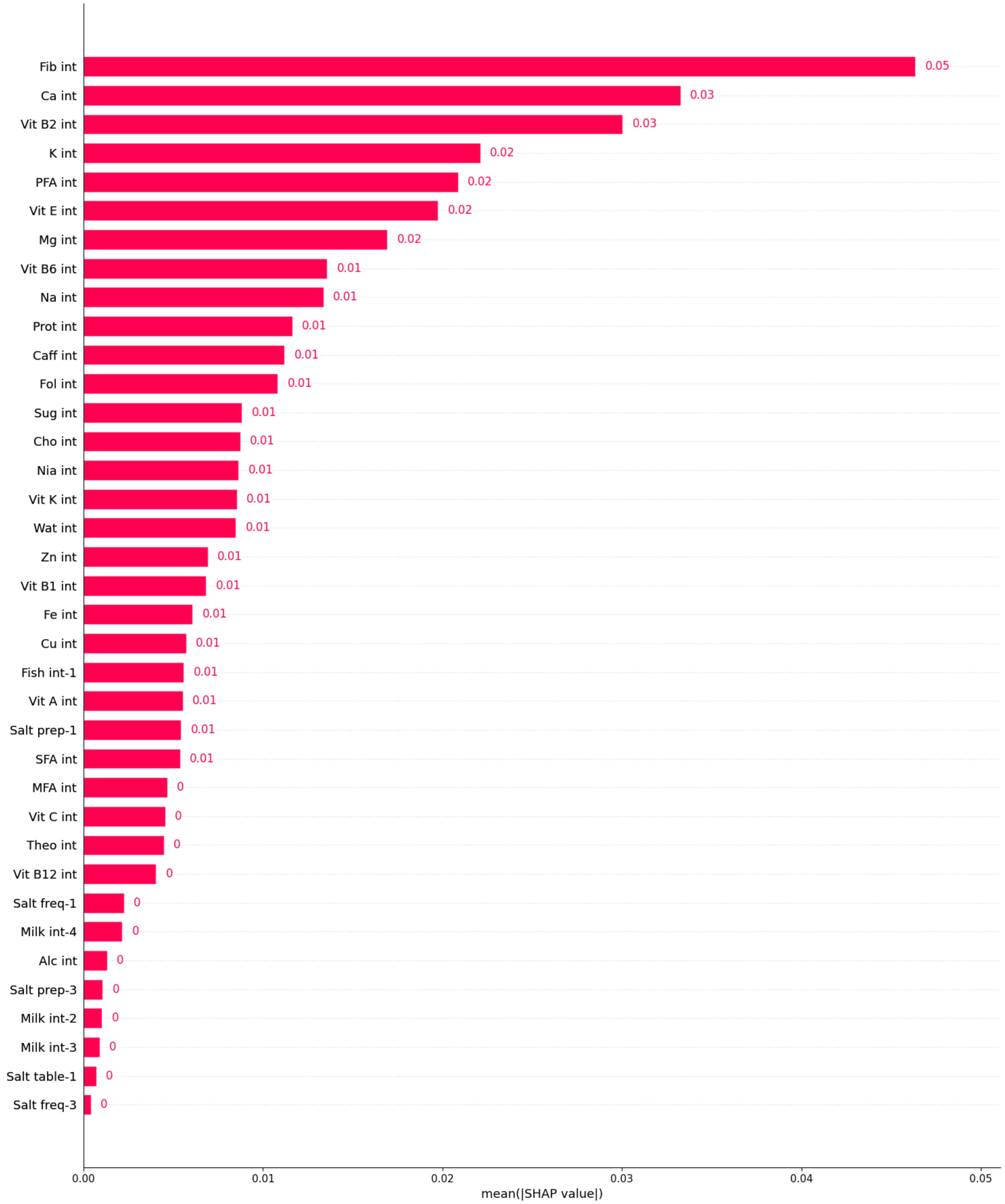

Figure S5. Feature importance from nutrition model for cardiovascular disease death based on SHAP absolute values.

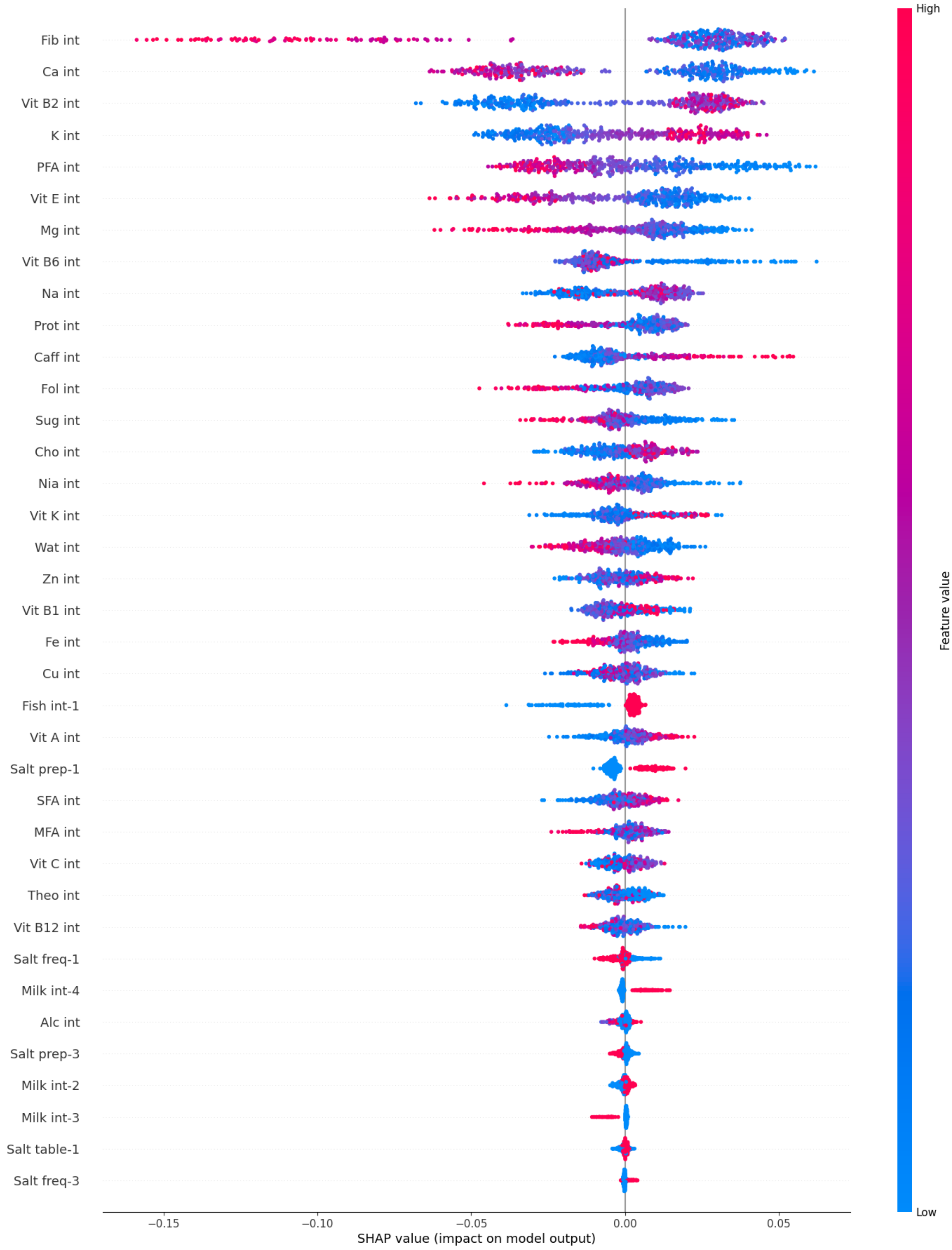

Figure S6. Feature importance from nutrition model for cardiovascular disease death based on SHAP value distribution. Positive SHAP-values are indicative of positive correlation with CVD death, while negative SHAP-values are indicative of negative correlation.
